# Long-Term Serological Follow-Up of Acute Q-Fever Patients after a Large Epidemic

**DOI:** 10.1371/journal.pone.0131848

**Published:** 2015-07-10

**Authors:** Cornelia C. H. Wielders, Joris A. F. van Loenhout, Gabriëlla Morroy, Ariene Rietveld, Daan W. Notermans, Peter C. Wever, Nicole H. M. Renders, Alexander C. A. P. Leenders, Wim van der Hoek, Peter M. Schneeberger

**Affiliations:** 1 Department of Medical Microbiology and Infection Control, Jeroen Bosch Hospital, ‘s-Hertogenbosch, the Netherlands; 2 Centre for Infectious Disease Control, National Institute for Public Health and the Environment (RIVM), Bilthoven, the Netherlands; 3 Academic Collaborative Centre AMPHI, Department of Primary and Community Care, Radboud university medical center (Radboudumc), Nijmegen, the Netherlands; 4 Department of Infectious Disease Control, Municipal Health Service (GGD) Hart voor Brabant, ‘s-Hertogenbosch, the Netherlands; Texas A&M Health Science Center, UNITED STATES

## Abstract

**Background:**

Serological follow-up of acute Q-fever patients is important for detection of chronic infection but there is no consensus on its frequency and duration. The 2007–2009 Q-fever epidemic in the Netherlands allowed for long-term follow-up of a large cohort of acute Q-fever patients. The aim of this study was to validate the current follow-up strategy targeted to identify patients with chronic Q-fever.

**Methods:**

A cohort of adult acute Q-fever patients, diagnosed between 2007 and 2009, for whom a twelve-month follow-up sample was available, was invited to complete a questionnaire and provide a blood sample, four years after the acute episode. Antibody profiles, determined by immunofluorescence assay in serum, were investigated with a special focus on high titres of IgG antibodies against phase I of *Coxiella burnetii*, as these are considered indicative for possible chronic Q-fever.

**Results:**

Of the invited 1,907 patients fulfilling inclusion criteria, 1,289 (67.6%) were included in the analysis. At any time during the four-year follow-up period, 58 (4.5%) patients were classified as possible, probable, or proven chronic Q-fever according to the Dutch Q-fever Consensus Group criteria (which uses IgG phase I ≥1:1,024 to as serologic criterion for chronic Q-fever). Fifty-two (89.7%) of these were identified within the first year after the acute episode. Of the six patients that were detected for the first time at four-year follow-up, five had an IgG phase I titre of 1:512 at twelve months.

**Conclusions:**

A twelve-month follow-up check after acute Q-fever is recommended as it adequately detects chronic Q-fever in patients without known risk factors. Additional serological and clinical follow-up is recommended for patients with IgG phase I ≥1:512, as they showed the highest risk to progress to chronic Q-fever.

## Introduction

Q-fever is a bacterial zoonosis caused by *Coxiella burnetii*. Between 2007 and 2009, one of the largest documented Q-fever epidemics occurred in the Netherlands, which originated from dairy goat farms and caused over 3,500 notified cases [1].

After an episode of acute Q-fever, *C*. *burnetii* may persist intracellularly, causing progression to chronic infection. While acute Q-fever presents as febrile illness, pneumonia or hepatitis, chronic Q-fever mainly presents as endocarditis or vascular infections with a high morbidity and mortality [2]. Patients with resolving acute Q-fever reach peak antibody titres in the first months after infection [3, 4], in contrast to chronic Q-fever patients, who have persistent elevated antibody titres, specifically IgG phase I [5].

In the aftermath of the Dutch Q-fever epidemic, the focus shifted from diagnosing acute Q-fever patients to early identification and treatment of patients with chronic Q-fever [2]. Based on the literature, 0–5% of acute Q-fever patients are estimated to develop chronic Q-fever [6]. These figures lack accuracy as case definitions differ for both acute and chronic infections [6]. There is also considerable uncertainty about the time it takes to develop chronic Q-fever which ranges from months to years [7–9]. A contributing cause of this variation is the diagnostic delay, as it is difficult to diagnose chronic Q-fever.

Nonetheless, follow-up to detect chronicity after acute Q-fever is generally considered essential, but there is no consensus about optimal timing, frequency, duration and the cut-off level of antibody titres [6, 9–12].

To identify chronic Q-fever patients as early as possible, the Jeroen Bosch Hospital (JBH) in ‘s-Hertogenbosch, located in the centre of the Dutch epidemic, provided active serological follow-up to acute Q-fever patients at three, six, and twelve months after diagnosis [10, 13]. A four-year follow-up study was conducted (Q-HORT) to validate the routine follow-up strategy for detecting chronic Q-fever by comparing the serological results in the first year with those at four-year follow-up. The aim of this study was to: (1) validate this follow-up strategy targeted to identify patients with chronic Q-fever; (2) check whether there are any groups of patients that need follow-up later than twelve months because of the risk to progress to chronic Q-fever; and (3) identify factors associated with an increased IgG phase I titre at four-year follow-up.

## Methods

### Ethics statement

This study was approved by the Medical Ethical Committee Brabant (METC Brabant, reference NL35654.028.11) and the Internal Review Board of JBH. Written informed consent was obtained from all participants.

### Study population

All patients diagnosed with acute Q-fever in 2007, 2008, and 2009 at the Laboratory of Medical Microbiology of JBH (catchment area of approximately 550,000 persons) were contacted for follow-up four years after their initial diagnosis. One-year follow-up results for the 2007 and 2008 cohort are described by van der Hoek et al. [10].

### Acute Q-fever case definition

Suspected Q-fever patients were referred by a general practitioner (GP) or a hospital physician for laboratory confirmation of the presumptive diagnosis of acute Q-fever. Diagnostic blood samples and samples at three and six months were used for identification of acute cases and confirmation of the diagnosis. A laboratory-confirmed acute Q-fever case was defined as either one of the following three criteria: (1) both IgM and IgG phase II antibody titres ≥1:32 in the diagnostic sample by immunofluorescence assay (IFA; Focus Diagnostics, Inc., Cypress, CA, USA) with IgG phase II ≥1:64 during follow-up; (2) enzyme-linked immunosorbent assay (ELISA; Virion\Serion, Würzburg, Germany) IgM phase II positive and IFA IgG phase II ≥1:32 at diagnosis with IgG phase II ≥1:64 during follow-up; (3) a positive polymerase chain reaction (PCR; in-house assay [14]) result preceding seroconversion in IFA (Focus Diagnostics, Inc.) with IgG phase II ≥1:64 during follow-up.

### Laboratory methods

ELISA IgM phase II and IFA IgM and IgG phase I and II were performed on diagnostic samples following the manufacturer’s instructions. IFA titres of ≥1:32 were considered positive. An in-house PCR test was performed when the sample was taken ≤14 days after onset of illness. The details of the PCR in-house assay targeting the IS*1111* insertion element have been described elsewhere [14]. IFA IgG phase I and II tests were performed in all three-, six-, and twelve month follow-up samples using two-fold dilutions starting at 1:32. An additional PCR test was performed when there was suspicion of chronic Q-fever. Diagnostic evaluations for patients suspected of having acute Q-fever were performed according to the diagnostic algorithm that was established in our laboratory in 2009 [15].

### Data collection

Serological data at diagnosis and at three-, six-, and twelve-month follow-up were available from the laboratory information system. Four years after diagnosis, a study information pack was sent by post which contained: an invitation for participation in this study, a questionnaire, an informed consent form, a diagnostic request form and leaflets showing laboratory locations. Patients were asked to visit a laboratory facility in their neighbourhood to have a blood sample taken. The signed informed consent form and questionnaire were to be returned by post. Patients who did not respond within four weeks were sent all of the study materials a second time.

The questionnaire consisted of questions on general demographics and risk factors for chronic Q-fever. Participants who failed to complete all of the questions in the questionnaire were subsequently contacted (by email, phone, or post). Answers were entered into IBM SPSS Statistics for Windows version 19.0.0 (SPSS Inc., Armonk, NY, USA) and 5% of the questionnaires were double-checked.

When participants reported that they had consulted a physician for cardiovascular problems, the hospital information systems of the two hospitals in the catchment area were checked for specific information on the condition (with the consent of the participants). The individual prescription and use of antibiotics was not available for most of the patients.

The blood samples were used to determine IgM and IgG phase I and II in serum using IFA. PCR was performed when IgG phase I was ≥1:512. Both participants and their GPs received the laboratory results.

### Chronic Q-fever case definition

Participants with IgG phase I ≥1:1,024 were categorized as proven, probable, or possible chronic Q-fever patients according to the Dutch Q-fever Consensus Group criteria [16]. Proven chronic Q-fever is defined as positive *C*. *burnetii* PCR (or culture) in blood or tissue (in the absence of acute infection), or a high IgG phase I titre (≥1:1,024 for commercial IFA) in combination with confirmed endocarditis according to the revised Duke criteria [17], or evident infection of aneurysm or vascular graft by imaging techniques. Probable chronic Q-fever patients have an IgG phase I ≥1:1,024, in combination with risk factors for chronic Q-fever, echocardiographic abnormalities that do not meet the revised Duke criteria [17], rare manifestations of Q-fever or signs of systemic inflammation. Possible chronic Q-fever cases have an IgG phase I ≥1:1,024, and do not have any of the manifestations mentioned in the categories of proven and probable Q-fever.

At the twelve-month follow-up of this cohort, these criteria were not yet established and an IgG phase I ≥1:2,048 was used to diagnose chronic Q-fever and to refer patients for clinical evaluation.

### Exclusion criteria

Patients without a serum sample obtained twelve months after diagnosis and those younger than 18 years at the four-year follow-up were excluded from this study. Patients were excluded for analysis when: (i) the date of onset of symptoms was uncertain; (ii) proven chronic Q-fever or a probable chronic infection was identified in the first blood sample that was submitted to the laboratory (i.e., no sample available or taken for an acute *C*. *burnetii* infection); (iii) at least IgM and/or IgG phase I/II antibodies were detected in the first serum sample or the PCR result was positive, but the IgG phase II titre was ≤1:32 in all follow-up samples (i.e., thus not meeting IgG phase II ≥1:64 during follow-up which is part of the case definition for a laboratory-confirmed case).

### Statistical analysis

For descriptive characteristics, relative frequencies were calculated with median and interquartile ranges (IQRs). Chi-square tests and Mann-Whitney U tests were used to test for differences between participants and non-responders. A *p*-value <0.05 was considered as statistically significant. The Wilcoxon signed rank test was used to test for differences between the twelve-month and four-year follow-up samples.

Demographic information and medical conditions present during the four-year follow-up study were used in a univariable and multivariable logistic regression analysis to find factors associated with an IgG phase I ≥1:512 at four-year follow-up (index: IgG phase I ≥1:512, reference: IgG phase I <1:512). For this analysis, heart valve abnormalities were defined as a prosthetic valve, grade ≥2 valve stenosis or regurgitation, mitral valve prolapse, bicuspid valve or other congenital cardiopathies, remodelling or thickening of the valve [18, 19]. Results are expressed as an odds ratio (OR) with a 95% confidence interval (95% CI). Risk factors which had a *p*-value <0.20 at the univariable level were selected for a conditional backward step-wise multivariable model. The significance level was set to 0.05 for the latter model. To assess the goodness-of-fit of the final model, a receiver operating characteristic (ROC) curve was produced and the area under the curve (AUC) was estimated.

Univariable and multivariable analyses were also performed with IgG phase I ≥1:256 and ≥1:1,024 used as cut-off titres. The analyses were repeated using a proportional odds ratio model, which enables logistic regression to be generalized to ordinal outcomes (titres), as it uses the whole spectrum of observations [20]. The proportional odds assumption is that the odds of being above any cut-off is the same for all cut-offs, so a single OR is calculated. This analysis has a greater power than binary logistic regression when ordinal data are dichotomized, and may identify risk factors that would remain undetected in binary logistic regression [20]. Analyses were performed using IBM SPSS Statistics and R version 3.0.1 (The R Foundation for Statistical Computing, Vienna, Austria).

## Results

### Inclusion and response

**Fig 1:**
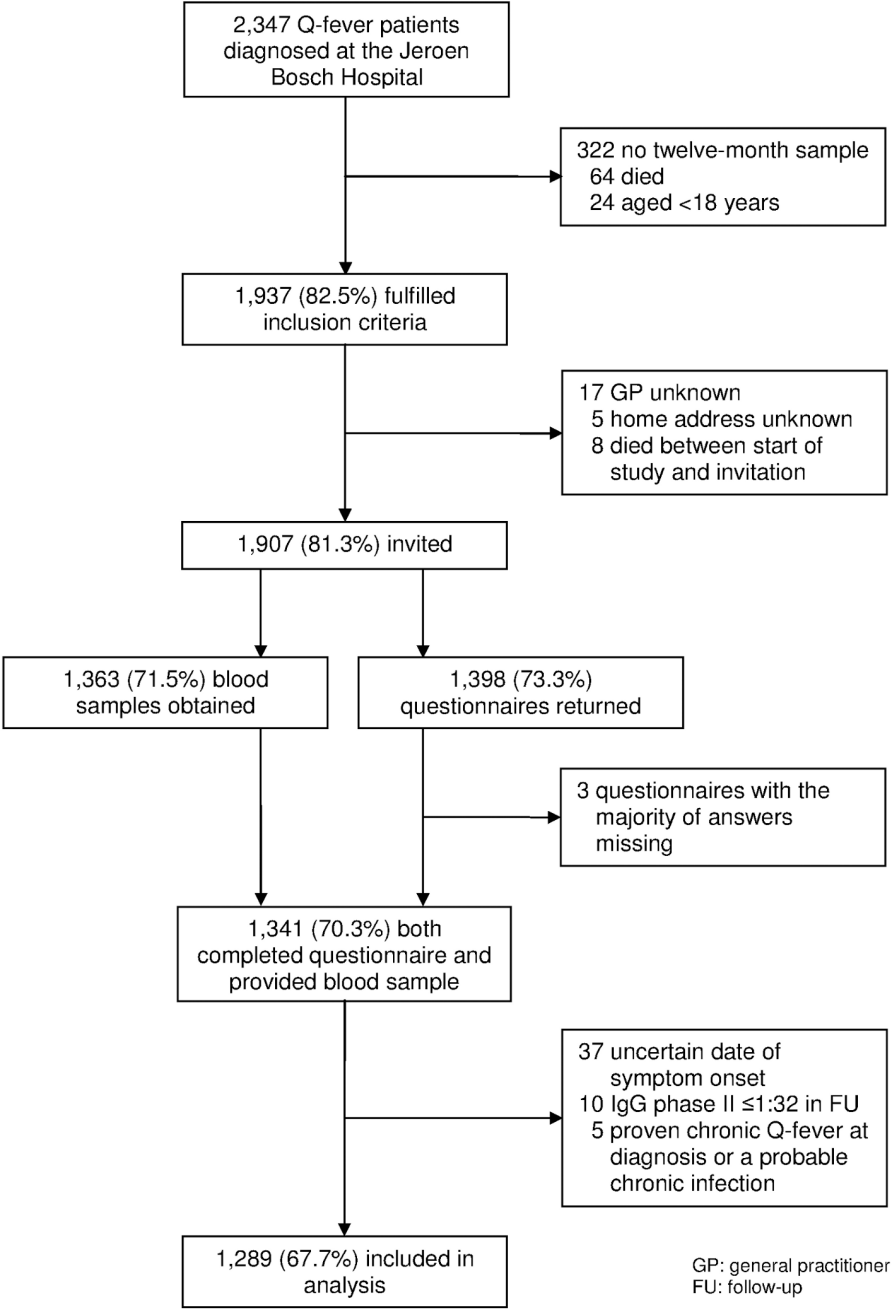
Flow chart of the four-year follow-up study (Q-HORT).

From 2007 to 2009, 2,347 Q-fever diagnoses were made at the Laboratory of Medical Microbiology of the JBH, of whom 1,937 (82.5%) fulfilled the study inclusion criteria, and 1,907 (81.3%) were invited (Fig 1 and Table 1). A complete questionnaire and blood sample were obtained from 1,341 patients. A total of 52 patients were additionally excluded for analysis (Fig 1), and 1,289 participants remained, 703 (54.5%) males, with a median age at diagnosis of 51 years (IQR 41−59).

**Table 1:** Response at four-year follow-up: received questionnaires and blood samples for each year of diagnosis and total numbers.

Year of diagnosis (year of invitation)	Fulfilled inclusion criteria	Invited	Blood sample	Questionnaire	Blood sample and questionnaire
	n	n	n (%)^a^	n (%)^a^	n (%)^a^
2007 (2011)	68	66 (97.1)	50 (75.8)	50 (75.8)	50 (75.8)
2008 (2012)	673	664 (98.7)	470 (70.8)	488 (73.5)	466 (70.2)
2009 (2013)	1,196	1,177 (98.4)	843 (71.6)	860 (73.1)	828 (70.3)
**Total**	1,937	1,907 (98.5)	1,363 (71.5)	1,398 (73.3)	1,344 (70.5)^b^

^a^ Percentages calculated with number of invited as denominator.

^b^ Including three questionnaires with the majority of answers missing (Fig 1).

A total of 330 (17.9%) patients did not respond to our invitations to participate in the study. Just 150 (7.9%) stated that they did not want to participate, and 10 had died between the twelve-month and four-year follow-up, a fact that was not known to the investigators at the time of sending the invitations. Characteristics of patients who were included in the analysis (n = 1,289) were compared to those who were excluded (meaning those who fulfilled the exclusion criteria, who did not participate, or who participated only partially (n = 648; 1,937 participants fulfilling inclusion criteria minus 1,289 participants included in the analysis)). Excluded patients were statistically significantly more often male and younger at diagnosis than participants who were included for analysis (male: 62.2% vs. 54.5%, *p* = 0.001; median age: 45 vs. 51 years, *p*<0.001), but antibody levels at diagnosis, three-, six, and twelve-month follow-up, the geographic location (postal code), and hospitalization for acute Q-fever were not significantly different between these two groups.

### Chronic Q-fever detected during four-year follow-up

At any time during the four-year follow-up period, 58 (4.5%) patients could be classified as possible, probable, or proven chronic Q-fever (Table 2). The majority of these (52 or 89.7%) had already been identified within the first year after the acute episode when applying the Dutch Q-fever Consensus Group criteria [16]. Of the six patients that were detected at four-year follow-up, five had an IgG phase I of 1:512 at twelve months (5 of a total of 73 individuals with IgG phase I of 1:512 at month twelve), while one patient had a titre of 1:128 at twelve months. These six patients were classified as one proven, one probable, and four possible, chronic Q-fever patients (Table 3). The probable chronic Q-fever patient detected at four-year follow-up did not have fever, pneumonia, or hepatitis at the time of the primary *C*. *burnetii* infection. Furthermore, three of the 52 patients identified within the first year showed progression to probable or proven chronic Q-fever at the four-year follow-up (Table 2). Fifteen patients were identified at any time during the four-year follow-up period with a probable or proven chronic Q-fever (1.2% of all participants).

**Table 2:** Chronic Q-fever patients detected at any time during the four-year follow-up period according to the Dutch Q-fever Consensus Group classification [16] based on IgG phase I ≥1:1,024 (n = 58).

Chronic Q-fever	Proven	Probable	Possible	Total
	n	n	n	n
Identified within the first year^a^	4	7	41	52
Identified at four-year follow-up^b^	1	1^c^	4	6
Progression detected at four-year follow-up^d^	2^e^	1^f^	NA^g^	NA

^a^ The Dutch Q-fever Consensus Group classification [16] was not established yet at twelve-month follow-up, and IgG phase I ≥1:2,048 was used at that time to diagnose chronic Q-fever. For this table, we applied the criteria of the Dutch Q-fever Consensus Group (IgG phase I ≥1:1,024) to the data.

^b^ These participants did not meet the criteria of the Dutch Q-fever Consensus Group [16] at twelve months: one possible chronic Q-fever patient had an IgG phase I titre of 1:128 at month twelve, the remaining five had a titre of 1:512 (Table 3).

^c^ This patient did not have fever, pneumonia, or hepatitis at time of the primary *C*. *burnetii* infection.

^d^ These patients are included in the number of chronic Q-fever cases identified within the first year.

^e^ One possible to proven (at time of visiting the outpatient clinic for our study the newly obtained serum sample was *C*. *burnetii* PCR positive), and one probable to proven (despite serological and clinical follow-up later than twelve months after diagnosis, the probable chronic infection progressed to a proven Q-fever endocarditis that was detected based on our follow-up study).

^f^ One possible to probable (valvulopathy not meeting the modified Duke criteria [17]).

^g^ Two possible chronic Q-fever patients (identified at month twelve) were referred to the outpatient clinic at four-year follow-up, but no progression occurred.

**Table 3:** Clinical and serological findings of the six patients identified at four-year follow-up.

No.	IgG phase I twelve months	IgG phase I four years	PCR result	Clinical findings	Chronic Q-fever category
1	1:512	1:8,192	Negative	Immunosuppression and endocarditis^a^	Proven
2^b^	1:512	1:4,096	Negative	Aneurysm	Probable
3	1:512	1:1,024	Negative	No risk factors^c^	Possible
4	1:512	1:1,024	Negative	No risk factors^c^	Possible
5	1:512	1:1,024	Negative	No risk factors^c^	Possible
6	1:128	1:1,024	Negative	No risk factors^c^	Possible

^a^ Unknown whether risk factor (heart valve disease) was present in advance.

^b^ This patient did not have fever, pneumonia, or hepatitis at time of the primary *C*. *burnetii* infection.

^c^ No cardiac or vascular risk factors.

### Antibodies at four-year follow-up

**Fig 2:**
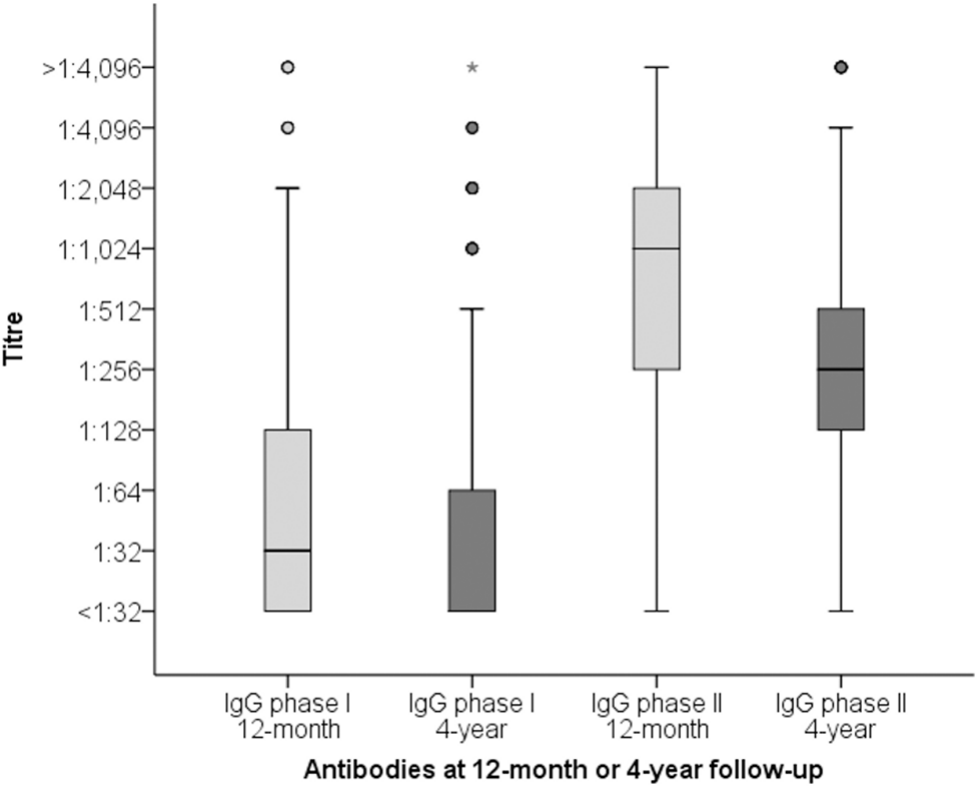
Boxplot of IgG phase I and II antibodies at twelve months and four years after acute Q-fever diagnosis (n = 1,289). The horizontal dark lines within the boxes represent the median antibody titre, the lower and upper boundaries of the boxes represent the 25^th^ and 75^th^ percentiles, and the T-bars represent the 2.5^th^ and 97.5^th^ percentiles. Outliers are indicated with dots, extreme outliers (more than three times the height of the box) with asterisks. Because there is less variation in the IgG phase I titres at four years compared to the other three boxes shown, this is the only one of the four boxes that shows extreme outliers.

Median IgG phase I and II antibody titres showed a statistically significant decline from month twelve to year four (*p*<0.001 for both IgG phase I and II; Fig 2), i.e. the median IgG phase I and II titres at four years (box 2 and 4, respectively) are lower than those at month twelve (box 1 and 3, respectively). Since IgG phase I ≥1:1,024 is used as a cut-off for chronic infection, it can be seen that some of the chronic Q-fever patients at month twelve (11 out of 52) and all chronic patients at year four are shown as outliers.

**Fig 3:**
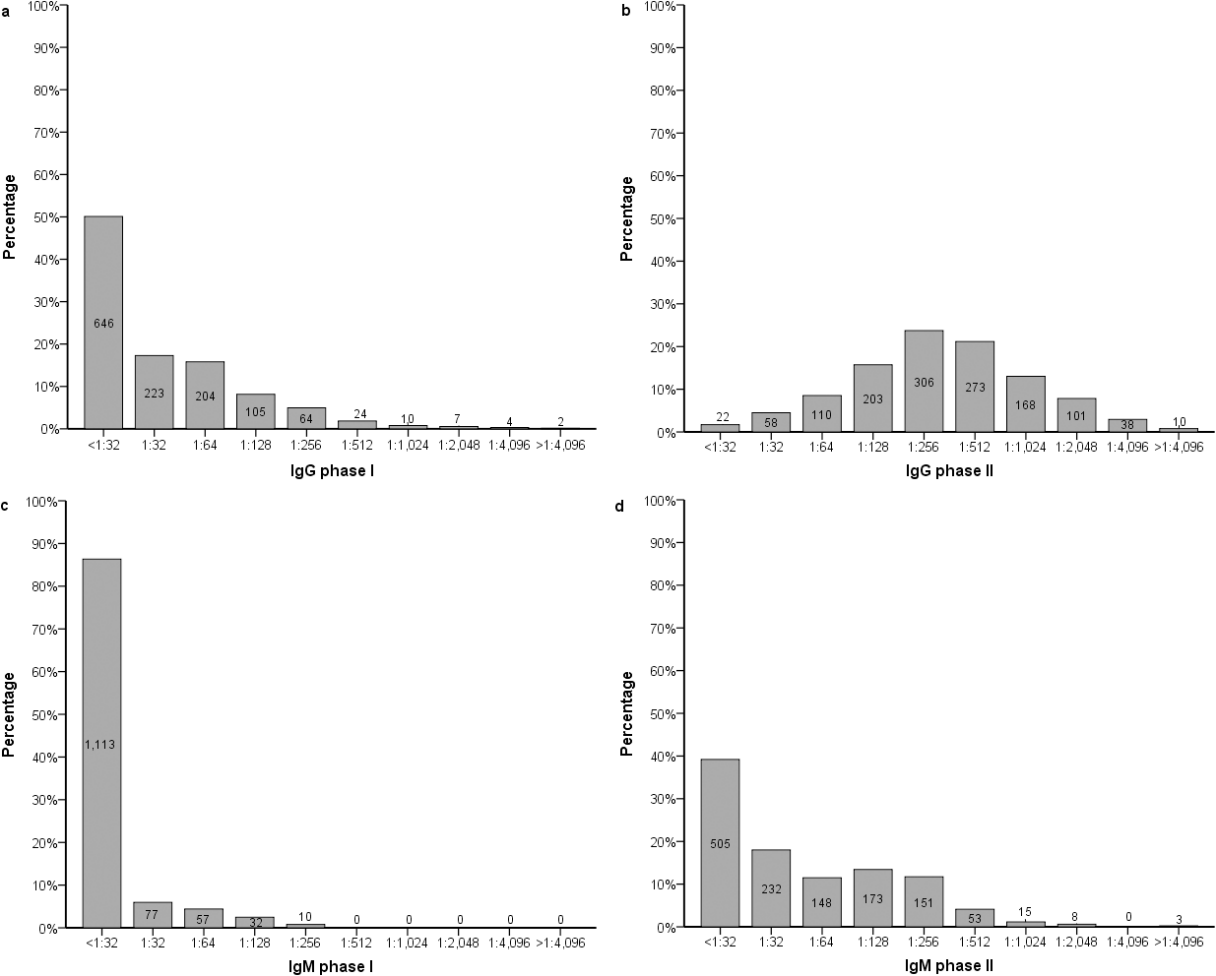
Bar charts of IgG phase I (a), IgG phase II (b), IgM phase I (c), and IgM phase II (d) antibody levels against Coxiella burnetii, four years after acute Q fever episode (n = 1,289). All 47 samples with an IgG phase I titre ≥1:512 had a negative *C*. *burnetii* PCR result. The numbers presented in the bars indicate the number of patients.

At four-year follow-up, IgG phase II had the highest titres and was detected in almost all participants (Fig 3). IgM phase II was also still detectable in the majority of participants. Only 14 participants (1.1%) had no detectable antibody levels at four years. An increase in IgG phase I of two or more dilutions at four-year follow-up was found in 45 (3.5%) patients, of whom 10 had IgG phase I ≥1:64 at month twelve (S1 Table).

### Risk factor analysis

In univariable analysis, age >55 years, aneurysm, vascular prosthesis, heart valve insufficiency or prosthesis, percutaneous coronary intervention (PCI), coronary stent, rheumatoid arthritis (RA), and immunosuppressive medication were significantly associated with an IgG phase I ≥1:512 at four-year follow-up (*p*<0.05) (Table 4). In multivariable analysis, age >55 years, aneurysm, PCI, and RA remained significantly associated with an IgG phase I ≥1:512 at four-year follow-up (AUC 0.68, 95% CI: 0.60−0.76). Known cardiovascular risk factors for chronic Q-fever (heart valve defect or prosthesis, vascular prosthesis, or aneurysm), when combined, showed increased odds for an IgG phase I ≥1:512 at four-year follow-up (OR 4.64, 95% CI: 1.86−11.58). The proportional odds ratio analysis resulted in lower risk estimates with narrower 95% confidence intervals, but did not reveal any risk factors that were unidentified in binary logistic regression.

**Table 4:** Risk factors associated with an IgG phase I titre ≥1:512 at four-year follow-up.

Characteristic		IgG phase I ≥1:512 at four-year follow-up (index) (n = 47)	IgG phase I <1:512 at four-year follow-up (reference) (n = 1,242)	Univariable analysis			Multivariable analysis^a^		
		n (%)	n (%)	OR	95% CI	*p*	OR	95% CI	*p*
Male gender		28 (59.6)	675 (54.3)	1.24	0.68–2.24	0.481			
Age at four-year follow-up >55 years		32 (68.1)	586 (47.2)	2.39	1.28–4.45	0.005	1.94	1.05–3.76	0.036
Aortic aneurysm		2 (4.3)	4 (0.3)	13.76	2.46–77.07	<0.001	7.47	1.18–47.54	0.033
Vascular prosthesis		2 (4.3)	6 (0.5)	9.16	1.80–46.62	0.001			
Heart valve insufficiency / prosthesis^b^		4 (8.5)	31 (2.5)	3.63	1.22–10.75	0.020			
Myocardial infarction		2 (4.3)	55 (4.4)	0.96	0.23–4.06	0.955			
Coronary artery procedure	Coronary artery bypass surgery	2 (4.3)	22 (1.8)	2.47	0.56–10.80	0.216			
	Percutaneous coronary intervention	6 (12.8)	46 (3.7)	3.81	1.54–9.41	0.002	2.68	1.00–7.13	0.049
	Coronary stent	5 (10.6)	46 (3.7)	3.10	1.17–8.19	0.017			
Peripheral arterial procedure^c^		2 (4.3)	19 (1.5)	2.86	0.64–12.66	0.147			
Pacemaker		2 (4.3)	15 (1.2)	3.64	0.81–16.38	0.072			
Rheumatoid arthritis		10 (21.3)	92 (7.4)	3.38	1.63–7.01	0.001	3.10	1.47–6.53	0.003
Crohn’s disease / ulcerative colitis		0 (0.0)	14 (1.1)	NA	NA	NA			
Immunosuppressive therapy^d^	Intermediate suppressive	4 (8.5)	37 (3.0)	3.07	1.05–9.00	0.041			
	Severe suppressive	1 (2.1)	13 (1.0)	2.18	0.28–17.08	0.457			
Diabetes mellitus (type 1 and 2)		6 (12.8)	78 (6.3)	2.18	0.90–5.30	0.077			
Malignancy		4 (8.5)	75 (6.0)	1.45	0.51–4.14	0.488			
Chronic renal disease		0 (0.0)	18 (1.4)	NA	NA	NA			
Asthma		0 (0.0)	41 (3.3)	NA	NA	NA			
COPD		2 (4.3)	34 (2.7)	1.58	0.67–6.78	0.535			
Organ transplantation		0 (0.0)	3 (0.2)	NA	NA	NA			
Pregnancy in last 5 years^e^		1 (2.1)	34 (2.7)	0.77	0.10–5.77	0.801			

95% CI: 95% confidence interval; COPD: chronic obstructive pulmonary disease; NA: not applicable; OR: odds ratio.

^a^ Only factors significantly (*p*<0.05) associated in multivariable analysis are presented. Variables included at the start of the multivariable analysis: age >55 years, aneurysm, vascular prosthesis, heart valve insufficiency or prosthesis, percutaneous coronary intervention, pacemaker, peripheral arterial procedure, rheumatoid arthritis, immunosuppressive therapy, and diabetes mellitus. Area under the curve of final model: 0.68 (95%CI: 0.60–0.76).

^b^ Heart valve abnormalities included: having a prosthetic valve, grade ≥2 valve stenosis or regurgitation, mitral valve prolapse, bicuspid valve or other congenital cardiopathies, remodelling or thickening of the valve [18, 19]. Reference category is no reported valvular disease or only subtle or minor abnormalities not meeting our definition of heart valve disease.

^c^ Peripheral artery bypass, angioplasty or stent.

^d^ Immunosuppressive therapy included cytostatic treatment for cancer, immunosuppressive medication for kidney transplantation, inflammatory bowel disease, rheumatoid arthritis, etc. (e.g. biologicals, high doses of prednisolone (>10 mg/day for >2 weeks) and other corticosteroids). A distinction is made between intermediate and severe immunosuppression. Intermediate immunosuppressive therapy: oral/rectal administered immunosuppressive drugs; severe immunosuppressive therapy: subcutaneous/intravenous administered immunosuppressive drugs (including cytostatic treatment) and oral cytostatic treatment.

^e^ Calculated for women only.

## Discussion

The most important finding of this four-year follow-up study is that only one possible chronic Q-fever patient was detected who was not already identified as being at risk at twelve-month follow-up. This patient had no clinical risk factors and the IgG phase I was only 1:128 at twelve-month follow-up. Another five patients with newly detected chronic Q-fever at four-year follow-up had an IgG phase I of 1:512 at twelve months. When combined with the 52 chronic Q-fever patients that had already been detected within the first year, this means that determination of IgG phase I antibody titre at twelve months with a 1:512 cut-off for further clinical investigation, detects 98% of people at risk for developing chronic Q-fever (IgG phase I ≥1:1,024 [16]).

Several guidelines exist on the follow-up of acute and chronic Q-fever patients. The Centres for Disease Control and Prevention (CDC) published guidelines for both acute and chronic Q-fever [11], the Dutch Q-fever Consensus Group focussed on chronic Q-fever [16], and also individual reports gave advice on follow-up [9, 10, 12]. Our follow-up advice deviates at some points from these published guidelines and recommendations. First, serological follow-up after three months is, in our opinion, too soon for acute Q-fever patients without known risk factors, as IgG titres can still increase [4, 10]. Previous studies have been inconclusive in this respect [4, 9, 10, 12]. Secondly, for the majority of patients with IgG phase I ≥1:1,024 at six months we observed a decline in IgG phase I titres at twelve months (data not shown). This implies that when serological follow-up is performed at six months, and clinical evaluation is needed for patients with increased IgG phase I titres [11], a large number of patients would undergo these clinical evaluations unnecessarily.

**Fig 4:**
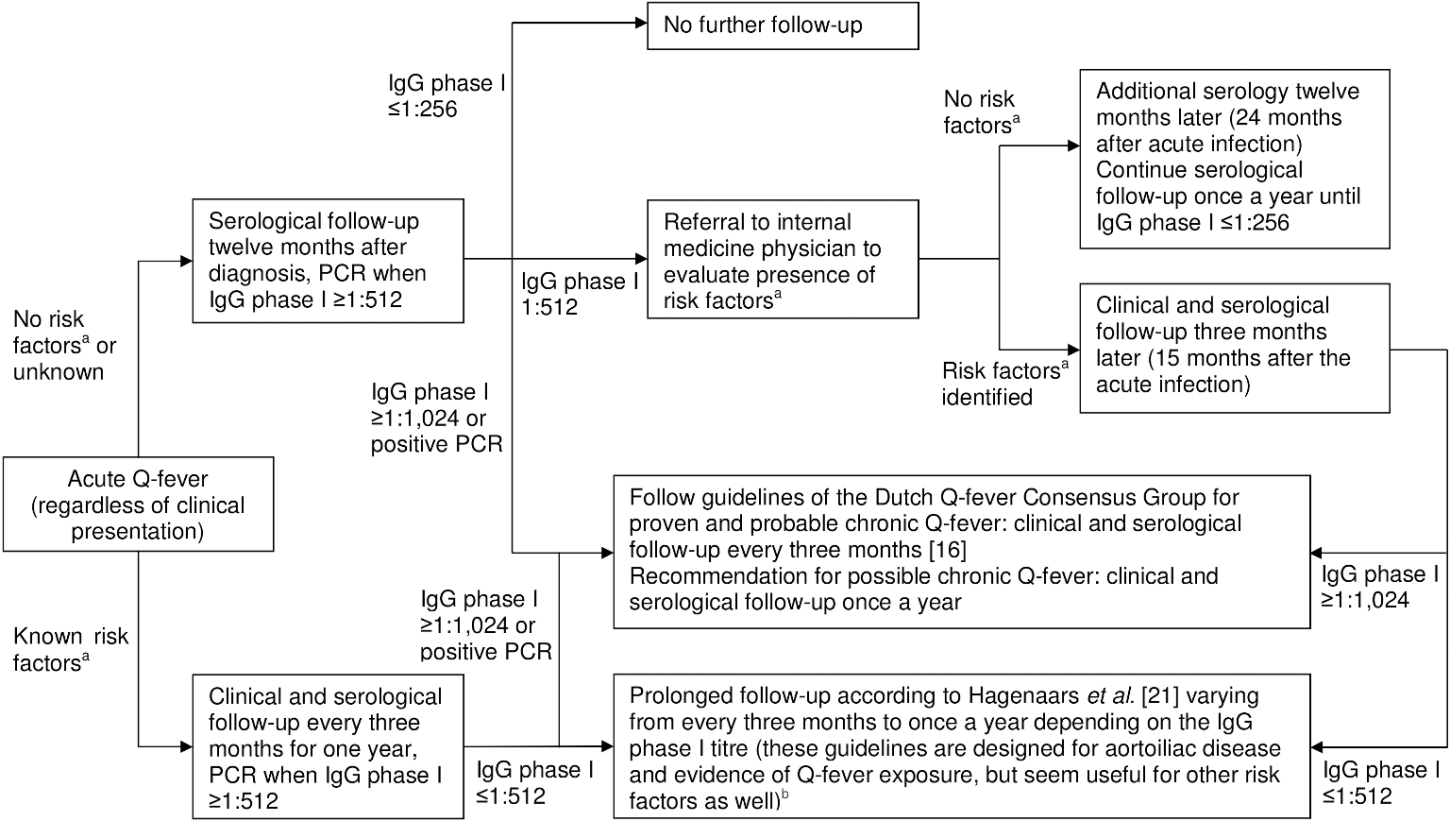
Recommendations for serological and clinical follow-up in acute Q-fever patients regardless of compatible clinical presentation. ^a^ Heart valve/vascular disease or prosthesis. ^b^ See [21].

Therefore, based on our data, we propose serological follow-up after twelve months in patients who do not have risk factors (Fig 4). Finally, in the absence of risk factors, patients with IgG phase I ≥1:512 at twelve-month follow-up, including all possible chronic Q-fever patients (IgG phase I ≥1:1,024 without any clinical sign of chronic Q-fever), can be considered to have a low risk of developing proven chronic Q-fever within four years. With these newest insights, we feel that regular checks every three months for possible chronic Q-fever patients, as recommended by the Dutch Q-fever Consensus guidelines [16], can be replaced by an annual follow-up.

The number of studies investigating long-term serological follow-up after Q-fever are scarce, and recommendations about timing, frequency, and duration of follow-up to rule out chronic Q-fever are often inconsistent [6]. As far as we know, this is the largest epidemic cohort with four-year follow-up data ever reported, and it will be difficult to replicate in the absence of a new larger Q-fever epidemic. Moreover, all patients were diagnosed in one laboratory facility.

In a large epidemic such as the one that occurred in the Netherlands, it is advisable to actively invite patients for serological follow-up instead of passive follow-up requests, as large differences have been observed in follow-up rates between both systems [13]. Nevertheless, patients with an asymptomatic *C*. *burnetii* infection can also progress to chronic infection [22], and therefore screening of risk groups (people with heart valve disease, vascular prosthesis or aneurysm) is useful during an epidemic [6, 23, 24].

For chronic and acute Q-fever, many different case definitions are, or have been used in the literature [6]. Four years after the acute Q-fever diagnosis, a large number of patients in the present study still had detectable IgM phase II antibodies, which is considered a marker of acute infection. This is in contrast to earlier studies that showed undetectable levels within four months or low levels after eleven months [3, 25]. While presence of IgM phase II is an important diagnostic criterion for acute Q-fever in low-endemic areas, such as the Netherlands at the time of the first outbreak in 2007, its relevance is questionable in late epidemic and endemic situations because it can also indicate past-resolved Q-fever [26].

The major established risk factors for chronic Q-fever are valvular disease or surgery, vascular prostheses, and aneurysms [22, 27–29]. Heart valve and vascular endothelium is the preferred localization of chronic Q-fever. Age has also been reported as a risk factor, and probably reflects the increased prevalence of cardiovascular diseases and decreased cellular immunity during aging [10, 22, 30, 31]. We identified age, aneurysm, PCI, and RA as risk factors for an IgG phase I titre ≥1:512 at four-year follow-up. The use of one cut-off, however, is arbitrary, and therefore we performed two additional cut-off analyses (1:256 and 1:1,024) and a proportional odds ratio analysis. The risk factors identified in the IgG phase I ≥1:512 analysis were not confirmed as major risk factor in the additional analyses. This suggests that, besides serological follow-up for high-risk patients, routine serological follow-up for all other acute Q-fever patients at twelve months is important.

The individual prescription and use of antibiotics was not recorded for most of the patients. Previous studies, performed by several authors of the current study, have investigated the role of antibiotic therapy on hospitalization and antibody responses [32, 33]. Both studies included subjects of the present study. Dijkstra *et al*. showed that the majority of their study population, consisting of acute Q-fever patients diagnosed in 2007 or 2008, received adequate antibiotic treatment (doxycycline, 200 mg/day or moxifloxacin): 60% in 2007, increasing to 72% in 2008 [32]. Because of increasing awareness among physicians in the consecutive years, the number of patients receiving proper antibiotic treatment is expected to be higher in 2009 than in 2007. This was confirmed by a questionnaire study among general practitioners performed in 2009, which showed that 95% of the GPs started antibiotic treatment in patients with pneumonia of fever without awaiting the laboratory confirmation [34], and by a study of Wielders *et al*. [33]. This latter study investigated the effect of early diagnosis and start of treatment on the IgG antibody response in patients diagnosed in 2009. Adequate antibiotic treatment was prescribed in 83% (defined as at least 10 days of doxycycline (200 mg/day), moxifloxacin (400 mg/day), ciprofloxacin (1,000 mg/day) or cotrimoxazole (1,920 mg/day)). Early diagnosis and treatment did not prohibit the antibody response up to one year after the acute Q-fever diagnosis [33].

The initial self-reporting of underlying medical conditions and medication use represented a limitation of our study. For those participants who reported cardiovascular problems, we checked the hospital information system to confirm and categorize the conditions. We were unable to perform this check for all reported underlying diseases, since not all patients had hospital records and their underlying diseases were only known by their GP. This difference in quality assessment of medical conditions is a limitation of this study. Furthermore, we do not know if all patients reported all their medical treatments. Another limitation is that a more in-depth analysis of the participants versus non-participants was not possible, because no detailed data were available except age, sex, geographical location, hospitalization for acute Q-fever, and antibody titres. The majority of the patients that did not want to participate stated that they were not interested in the study, had no time to participate, had been too often invited for research, or because of reasons or a disease that is not related to Q-fever. Nevertheless, the influence of non-participation might have caused some bias or affected the representativeness.

## Conclusions

We recommend a single serological follow-up at twelve months after diagnosis for laboratory-confirmed acute Q-fever patients without known risk factors for chronic Q-fever (heart valve/vascular disease or prosthesis), regardless of a compatible clinical presentation during the acute infection (Fig 4). Further serological and clinical follow-up is required for patients without risk factors but with IgG phase I ≥1:512 after twelve-month follow-up. We suggest this should be done on an annual basis until IgG phase I is ≤1:256. Serological and clinical follow-up should be carried out more frequently, for example every three months, for patients with known risk factors. For patients with probable or proven chronic Q-fever, we suggest that the guidelines of the Dutch Q-fever Consensus Group are followed.

## Supporting Information

S1 Appendix Questionnaire used for the Q-HORT study (in Dutch). (DOC)

S1 Table Changes in IgG phase I antibody titres between twelve-month and four-year follow-up. (DOCX)
