# Supplementary material for: Long-Term Serological Follow-Up of Acute Q-Fever Patients after a Large Epidemic
Source: PLoS One. 2015 Jul 10;10(7):e0131848. doi: 10.1371/journal.pone.0131848 (PMC4498618; doi:10.1371/journal.pone.0131848)
Supplement: S1 Appendix — (DOC) [file pone.0131848.s001.doc]

# Onderzoek naar de mogelijke gevolgen

# van Q-koorts op de lange termijn

# Vragenlijst

Dit onderzoek wordt uitgevoerd door:


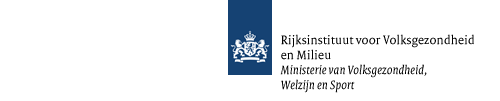


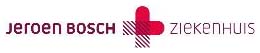


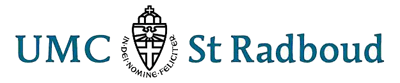


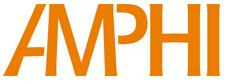

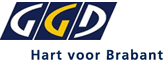


**Inleiding**

Sinds 2007 hebben veel mensen Q-koorts gehad. De meeste mensen zijn volledig hersteld maar anderen houden nog lange tijd klachten zoals moeheid. Bij een klein deel (ongeveer 2%) ontstaat chronische Q-koorts wanneer de Q-koorts bacterie in het lichaam aanwezig blijft. Uit eerder onderzoek is naar voren gekomen dat bepaalde aandoeningen een risico kunnen vormen voor chronische Q-koorts.

Wij hebben u gevraagd mee te doen aan een onderzoek waarvoor een bloedafname nodig is en het invullen van deze vragenlijst. Om de uitslag van het bloedonderzoek goed te kunnen begrijpen is het van groot belang dat u deze vragenlijst over uw algemene gezondheidstoestand en onderliggende aandoeningen invult.

# Opbouw van de vragenlijst

Deze vragenlijst bestaat uit 14 pagina’s. Eerst worden 4 algemene vragen gesteld. Dan volgen 46 vragen over uw (algemene) gezondheidstoestand, gezondheidsproblemen, onderliggende aandoeningen en eventuele operaties.

**Hoe lang duurt het invullen?**

Het invullen van deze vragenlijst duurt maximaal 15 minuten.

**Privacy**

Uw persoonsgegevens (zoals naam en postcode) zullen alleen worden gebruikt om uw antwoorden in deze vragenlijst te koppelen aan uw bloeduitslag. Daarna worden deze gegevens verwijderd en worden dus niet meer gebruikt in het verdere onderzoek.

**Hoe vult u de vragenlijst in?**

Beantwoordt u s.v.p. alle vragen, tenzij anders vermeld. Lees de vragen goed door voordat u ze beantwoordt.

Er zijn verschillende antwoordmogelijkheden:

1. U kruist het hokje aan bij het antwoord dat voor u van toepassing is

of

1. U vult het antwoord in op de stippellijn of in de hokjes

Als u zich vergist met het aankruisen van het hokje, maakt u het foute hokje helemaal zwart en kruist u het goede vakje aan en zet er een pijltje bij (zie voorbeeld).

Voorbeeld:

U bent een vrouw en geboren op 25 maart 1969, dan vult u de vragen zo in:

Wat is uw geslacht?  Man *U heeft ingevuld dat u een man bent*

Vrouw

Man *Verbeterd: u heeft ingevuld dat u een vrouw bent*

Vrouw

Wat is uw geboortedatum?

**Vragen over het onderzoek of de vragenlijst**

Hebt u vragen over het onderzoek of de vragenlijst? Dan kunt u contact opnemen met één van de personen die vermeld staan in de bijgevoegde brief.

**Algemene informatie**

1. Wat is uw naam? …………………………………………………….…
2. Wat is uw geslacht?  Man

Vrouw

1. Wat is uw geboortedatum? - -

(dag) (maand) (jaar)

1. Wat is de postcode van uw huidige adres?

(cijfers) (letters)

**Gezondheidstoestand I**

*Aandoeningen aan de bloedvaten*

1. Bent u ooit onder behandeling geweest voor een aandoening aan uw bloedvaten? (spataderen of hoge bloeddruk niet meegerekend)

Ja

Nee 🡪 *ga door naar vraag 13*

1. Hebt u voor deze aandoening ooit een vaatchirurg of andere specialist bezocht?

Ja

Nee

1. Wanneer hebt u uw huisarts en/of specialist voor het eerst bezocht voor deze aandoening?

Vóór 2007

In 2007

In 2008

In 2009

In 2010

In 2011

In 2012

In 2013

Weet ik niet

Niet van toepassing

1. Is er ooit een verbreding van de buikslagader (aneurysma) bij u vastgesteld?

Ja

Nee 🡪 *ga door naar vraag 10*

1. Wanneer werd dit aneurysma vastgesteld?

Vóór 2007

In 2007

In 2008

In 2009

In 2010

In 2011

In 2012

In 2013

Weet ik niet

1. Bent u ooit geopereerd aan uw bloedvaten?

Ja

Nee 🡪 *ga door naar vraag 13*

1. Hebt u ooit de volgende operaties gehad of bloedvatprothesen gekregen?

*Kruis a.u.b. bij a t/m d ‘Ja’, ‘Nee’ of ‘Weet niet’ aan indien u een vaatprothese hebt gehad*

- 1. Buisprothese  Ja  Nee  Weet niet
  2. Broekprothese  Ja  Nee  Weet niet
  3. Vaatomleiding  Ja  Nee  Weet niet
  4. Andere vaatprothese, namelijk:……………………………………………………...

1. Wanneer vond deze operatie (buisprothese, broekprothese, vaatomleiding of andere vaatprothese) plaats?

Vóór 2007

In 2007

In 2008

In 2009

In 2010

In 2011

In 2012

In 2013

Weet ik niet

Niet van toepassing

*Aandoeningen aan het hart*

1. Bent u ooit onder behandeling geweest voor een aandoening aan een hartklep?

Ja

Nee 🡪 *ga door naar vraag 18*

1. Hebt u voor deze aandoening ooit een cardioloog, hartchirurg of andere specialist bezocht?

Ja

Nee

1. Wanneer hebt u uw huisarts en/of specialist voor het eerst bezocht voor deze aandoening aan de hartklep?

Vóór 2007

In 2007

In 2008

In 2009

In 2010

In 2011

In 2012

In 2013

Weet ik niet

Niet van toepassing

1. Bent u ooit geopereerd aan een hartklep?

Ja

Nee 🡪 *ga door naar vraag 18*

1. In welk jaar hebt u deze operatie ondergaan?

Vóór 2007

In 2007

In 2008

In 2009

In 2010

In 2011

In 2012

In 2013

Weet ik niet

1. Hebt u ooit:

*Kruis a.u.b. bij a t/m e ‘Ja’ of ‘Nee’ aan.*

- 1. Een hartaanval (hartinfarct) gehad?  Ja  Nee

- 1. Een dotter behandeling ondergaan?  Ja  Nee
  2. Een stent gekregen?  Ja  Nee
  3. Een bypass operatie ondergaan?  Ja  Nee
  4. Een pacemaker gekregen?  Ja  Nee

*Hart- en vaatziekten (algemeen)*

1. Bent u ooit onder behandeling geweest van een neuroloog in verband met een hersenbloeding, herseninfarct of beroerte (CVA)?

Ja

Nee

1. Komen hart- en vaatziekten voor bij één of meer familieleden van onder de 70 jaar (ouders, broers of zussen)? (hoge bloeddruk niet meegerekend)

Ja

Nee

Weet ik niet

*Transplantatie*

1. Hebt u ooit een transplantatie ondergaan?

Ja

Nee 🡪 *ga door naar vraag 24*

1. In welk jaar hebt u deze transplantatie ondergaan?

Jaar:

1. Wat voor transplantatie was dit?

……………………………………………………………………………………………………

……………………………………………………………………………………………………

*Kanker*

1. Is bij u in de afgelopen 5 jaar kanker vastgesteld?

Ja

Nee 🡪 *ga door naar vraag 27*

1. Welke vorm(en) van kanker en in welk jaar is dit vastgesteld?

1. …………...……………………………………………………sinds jaar:

2. …...……………………………………………………………sinds jaar:

1. Bent u nu nog steeds onder behandeling?

Ja

Nee, de laatste behandeling was in het jaar

*Andere chronische aandoeningen*

1. Hebt u één of meer van onderstaande aandoeningen die vastgesteld zijn door een arts?

*Kruis a.u.b. bij a t/m d ‘Ja’ of ‘Nee’ aan*

- 1. Reuma of reumatoïde artritis  Ja  Nee
  2. Colitis ulcerosa of ziekte van Crohn  Ja  Nee
  3. Diabetes (suikerziekte)  Ja  Nee
  4. Chronische nierziekte  Ja  Nee

1. Hebt u naast de ziekten die al aan bod zijn gekomen in deze vragenlijst nog één of meer andere chronische aandoeningen die vastgesteld zijn door een arts waarvoor u medicijnen krijgt?

Ja

Nee 🡪 *ga door naar vraag 30*

1. Om welke andere door een arts vastgestelde chronische aandoening(en) gaat het en sinds welk jaar hebt u deze aandoening(en)?

1. …………...……………………………………………………sinds jaar:

2. …...……………………………………………………………sinds jaar:

3. …...……………………………………………………………sinds jaar:

*Behandelingen*

1. Welke medicijnen hebt u de afgelopen 5 jaar regelmatig gebruikt?

1. …………...……………………………………………………………………………………

2. …...……………………………………………………………………………………………

3. …...……………………………………………………………………………………………

4. …………...……………………………………………………………………………………

5. …...……………………………………………………………………………………………

6. …...……………………………………………………………………………………………

1. Hebt u vanwege acute Q-koorts langer dan 1 maand antibiotica gebruikt?

Ja

Nee

Weet ik niet

1. De standaardbehandeling voor Q-koorts bestaat uit 2 of 3 weken antibiotica. Bent u gedurende de afgelopen jaren ook nog met een andere therapie behandeld voor Q-koorts?

Psychologische begeleiding

Cognitieve gedragstherapie

Begeleid bewegen bij fysiotherapeut (Graded Exercise Therapy)

Extra antibioticakuren

Overig, namelijk: ………………………………………………

Nee

1. Had u klachten ten tijde van uw acute Q-koortsinfectie?

Ja

Nee

Weet ik niet

*Werk*

1. Hoeveel uur per week doet u betaald werk?

Ik werk nu gemiddeld uren per week

34b.Hoeveel uur per week deed u betaald werk voordat u Q-koorts kreeg?

Ik werkte toen gemiddeld uren per week

1. Is het aantal uren dat u per week betaald werk uitvoert anders dan voor dat u ziek bent geworden door Q-koorts?

Ja, ik werk minder uren dan voorheen doordat ik Q-koorts heb gehad

Ja, het aantal uren dat ik werk is veranderd, maar dit komt niet door

Q-koorts

Nee, ik werk hetzelfde aantal uren als voor mijn ziekte

1. Kunt u op uw werk tijdens de uren dat u werkt weer dezelfde inzet geven als voordat u Q-koorts doormaakte?

Ja, want ik voel mij weer net zo goed als daarvoor 🡪 *ga door naar vraag 37*

Nee, ik kan mij niet 100% inzetten omdat ik:

Sneller moe ben

Mij niet kan concentreren

Anders………………………………………………….

36b.Komt het feit dat u zich nu niet meer 100% kan inzetten tijdens het werk volgens u door Q-koorts?

Ja

Nee

1. Bent u arbeidsongeschikt geworden nadat u Q-koorts doormaakte?

Nee, ik werd niet arbeidsongeschikt

Ja, ik werd arbeidsongeschikt maar dat had niets met Q koorts te maken

Ja, ik werd arbeidsongeschikt en dat komt volgens mij door Q-koorts

***De volgende 4 vragen zijn alleen bedoeld voor vrouwelijke deelnemers. Mannelijke deelnemers kunnen doorgaan bij het volgende onderdeel “Algemene gezondheidstoestand II”, vraag 42.***

1. Bent u in de afgelopen 5 jaar zwanger geweest en hoe vaak?

Ja, ik ben …... keer zwanger geweest in de afgelopen 5 jaar

Nee 🡪 *ga door naar het volgende onderdeel* *“Algemene*

*gezondheidstoestand II”, vraag 42*

1. Hebt u in de afgelopen 5 jaar een miskraam (of meerdere miskramen) gehad? Zo ja, hoe vaak?

Ja, ik heb …… keer een miskraam gehad

Nee

1. Had u andere medische problemen (complicaties) tijdens uw zwangerschap(pen)?

Ja

Nee 🡪 *ga door naar het volgende onderdeel* *“Algemene*

*gezondheidstoestand II”, vraag 42*

1. Welke complicatie(s) had u tijdens uw zwangerschap(pen)?

1. …………...……………………………………………………………………………………

2. …...……………………………………………………………………………………………

3. …...……………………………………………………………………………………………

**Algemene gezondheidstoestand II**

**INSTRUCTIE**

Zet bij iedere groep in de lijst hieronder een kruisje in het hokje achter de zin die het best past bij uw eigen gezondheidstoestand vandaag.

1. **Mobiliteit**

Ik heb geen problemen met lopen

Ik heb enige problemen met lopen

Ik ben bedlegerig

1. **Zelfzorg**

Ik heb geen problemen om mijzelf te wassen of aan te kleden

Ik heb enige problemen om mijzelf te wassen of aan te kleden

Ik ben niet in staat mijzelf te wassen of aan te kleden

1. **Dagelijkse activiteiten**

*(bijv. werk, studie, huishouden, gezins- en vrijetijdsactiviteiten)*

Ik heb geen problemen met mijn dagelijkse activiteiten

Ik heb enige problemen met mijn dagelijkse activiteiten

Ik ben niet in staat mijn dagelijkse activiteiten uit te voeren

1. **Pijn/klachten**

Ik heb geen pijn of andere klachten

Ik heb matige pijn of andere klachten

Ik heb zeer ernstige pijn of andere klachten

1. **Stemming**

Ik ben niet angstig of somber

Ik ben matig angstig of somber

Ik ben erg angstig of somber

1. Om mensen te helpen bij het aangeven hoe goed of hoe

9 0

8 0

7 0

6 0

5 0

4 0

3 0

2 0

1 0

100

Slechtst

voorstelbare

gezondheidstoestand

0

Best

voorstelbare

gezondheidstoestand

slecht een gezondheidstoestand is, hebben we een meetschaal

(te vergelijken met een thermometer) gemaakt. Op de meetschaal

hiernaast betekent “100” de beste gezondheidstoestand die u zich

kunt voorstellen, en “0” de slechtste gezondheidstoestand die u zich

kunt voorstellen.

We willen u vragen op deze meetschaal aan te geven hoe goed of

hoe slecht volgens u uw eigen gezondheidstoestand vandaag is.

Trek een lijn van het hokje hieronder naar het punt op de

meetschaal dat volgens u aangeeft hoe goed of hoe slecht uw gezondheidstoestand vandaag is.

**Uw gezondheidstoestand**

**vandaag**

48.De volgende 8 uitspraken gaan over moeheid in de afgelopen twee weken.

Het antwoord dat u geeft, geeft aan in welke mate u vindt dat de uitspraak op u

van toepassing is.

U kunt ook de tussenliggende hokjes gebruiken, om uw antwoord te nuanceren.

Ja, dat klopt Geen ja, geen nee Nee, dat klopt niet

1. Ik voel me moe.
2. Lichamelijk voel ik me

uitgeput.

1. Ik voel me fit.
2. Ik voel me slap.
3. Ik voel me uitgerust.
4. Lichamelijk voel ik me in een

Slechte conditie.

1. Ik ben gauw moe.
2. Lichamelijk voel ik me in een

uitstekende conditie.

49.Wat is uw hoogst genoten opleiding?

Geen

Lagere school

Lager beroepsonderwijs (bijv. LTS, LEAO)

Middelbaar algemeen voortgezet onderwijs (bijv. VMBO, MAVO, IVO, MULO)

Middelbaar beroeps onderwijs (bijv. MTS, MEAO, MHNO, INAS)

Hoger algemeen en voorbereidend wetenschappelijk onderwijs (bijv. HAVO, VWO, HBS, Gymnasium, Atheneum)

Hoger beroeps onderwijs (HBO)

Universiteit, wetenschappelijk onderwijs

1. Rookt u, en zo ja, hoeveel sigaretten rookt u gemiddeld per dag?

Ja, ik rook sigaretten per dag

Nee, ik ben sinds dagen / weken / maanden / jaren gestopt

*(doorhalen wat niet van toepassing is)*

Nee, ik heb nooit gerookt

Eventuele opmerkingen:

………………………………………………………………………………………………………

………………………………………………………………………………………………………

………………………………………………………………………………………………………

………………………………………………………………………………………………………

………………………………………………………………………………………………………

………………………………………………………………………………………………………

………………………………………………………………………………………………………

**Einde van de vragenlijst**

**Hartelijk dank voor uw medewerking!**
