# Supplementary material for: Long-Term Serological Follow-Up of Acute Q-Fever Patients after a Large Epidemic
Source: PLoS One. 2015 Jul 10;10(7):e0131848. doi: 10.1371/journal.pone.0131848 (PMC4498618; doi:10.1371/journal.pone.0131848)
Supplement: S1 Table — (DOCX) [file pone.0131848.s002.docx]

**S1 Table. Changes in IgG phase I antibody titres between twelve-month and four-year follow-up.**

| **IgG phase I antibody titre at twelve months** | **IgG phase I antibody titre at four years** | | | | | |
| --- | --- | --- | --- | --- | --- | --- |
|  | **Decreased ≥2 dilutions** | **Decreased 1 dilution** | **Unchanged** | **Increased 1 dilution** | **Increased ≥2 dilutions** | **Total** |
|  | **n (%)** | **n (%)** | **n (%)** | **n (%)** | **n (%)** | **n** |
| <1:32 | NA | NA | 383 (84.7) | 42 (9.3) | 27 (6.0) | 452 |
| 1:32 | NA | 136 (56.0) | 68 (28.0) | 31 (12.8) | 8 (3.3) | 243 |
| 1:64 | 81 (36.3) | 65 (29.1) | 48 (21.5) | 23 (10.3) | 6 (2.7) | 223 |
| 1:128 | 51 (37.5) | 45 (33.1) | 21 (15.4) | 17 (12.5) | 2 (1.5) | 136 |
| 1:256 | 60 (54.5) | 30 (27.3) | 15 (13.6) | 5 (4.5) | 0 (0.0) | 110 |
| 1:512 | 50 (68.5) | 12 (16.4) | 6 (8.2) | 3 (4.1) | 2 (2.7) | 73 |
| 1:1,024 | 20 (71.4)^a^ | 4 (14.3) | 2 (7.1) | 2 (7.1) | 0 (0.0) | 28 |
| 1:2,048 | 8 (61.5)^b^ | 1 (7.7) | 2 (15.4) | 2 (15.4)^c^ | 0 (0.0) | 13 |
| 1:4,096 | 5 (100.0)^d^ | 0 (0.0) | 0 (0.0) | 0 (0.0) | 0 (0.0) | 5 |
| ≥1:8,192 | 4 (66.7)^e^ | 1 (16.7) | 1 (16.7)^f^ | NA | NA | 6 |

NA: Not applicable.

^a^ One person received treatment for a proven chronic Q fever infection.

^b^ One person received treatment for a probable chronic Q fever infection.

^c^ One person received treatment for a probable chronic Q fever infection.

^d^ Two proven chronic Q fever patients: one received treatment and one currently receives treatment.

^e^ One person received treatment for a proven chronic Q fever infection.

^f^ One person received treatment for a probable chronic Q fever infection.
